# Thromboembolic complications among multiple injured patients with pelvic injuries: identifying risk factors for possible patient-tailored prophylaxis

**DOI:** 10.1186/s13017-021-00388-7

**Published:** 2021-08-26

**Authors:** Tim Kirchner, Rolf Lefering, Richard Sandkamp, Helge Eberbach, Klaus Schumm, Hagen Schmal, Jörg Bayer, TraumaRegister DGU

**Affiliations:** 1 Department of Orthopedics and Trauma Surgery, Medical Center - Albert-Ludwigs-University of Freiburg, Faculty of Medicine, Albert-Ludwigs-University of Freiburg, Hugstetter Str. 55, 79106 Freiburg, Germany; 2 IFOM - Institute for Research in Operative Medicine, University Witten/Herdecke, Faculty of Health, Ostmerheimer Str. 200, 51109 Köln, Germany; 3 Department of Orthopedic Surgery, University Hospital Odense, Sdr. Boulevard 29, 5000 Odense C, Denmark; 4 Committee on Emergency Medicine, Intensive Care and Trauma Management (Sektion NIS) of the German Trauma Society, Berlin, Germany

**Keywords:** Multiple trauma, Thromboembolic event, Injury Severity Score, Pelvic fracture, Acetabular fracture

## Abstract

**Background:**

Patients with pelvic and/or acetabular fractures are at high risk of developing thromboembolic (TE) complications. In our study we investigate TE complications and the potential negative effects of concomitant pelvic or acetabular injuries in multiple injured patients according to pelvic/acetabular injury severity and fracture classification.

**Methods:**

The TraumaRegister DGU® was analyzed between 2010 and 2019. Multiple injured patients with pelvic and/or acetabular fractures with ISS ≥ 16 suffering from TE complications were identified. We conducted a univariate and multivariate analysis with TE events as independent variable to examine potential risk factors and contributing factors.

**Results:**

10.634 patients met our inclusion criteria. The overall TE incidence was 4.9%. Independent risk factors for the development of TE complications were sepsis, ≥ 10 operative interventions, mass transfusion (≥ 10 PRBCs), age ≥ 65 years and AIS_Abdomen_ ≥ 3 (all *p* < 0.001). No correlation was found for overall injury severity (ISS), moderate traumatic brain injury, additional injury to lower extremities, type B and C pelvic fracture according to Tile/AO/OTA and closed or open acetabular fracture.

**Conclusions:**

Multiple injured patients suffering from pelvic and/or acetabular fractures are at high risk of developing thromboembolic complications. Independent risk factors for the development of thromboembolic events in our study cohort were age ≥ 65 years, mass transfusion, AIS_Abdomen_ ≥ 3, sepsis and ≥ 10 surgery procedures. Among multiple injured patients with acetabular or pelvic injuries the severity of these injuries seems to have no further impact on thromboembolic risk. Our study, however, highlights the major impact of early hemorrhage and septic complications on thromboembolic risk in severely injured trauma patients. This may lead to individualized screening examinations and a patient-tailored thromboprophylaxis in high-risk patients for TE. Furthermore, the number of surgical interventions should be minimized in these patients to reduce thromboembolic risk.

## Background

Thromboembolic (TE) events are relevant complications during the treatment of multiple injured patients. TE incidence among trauma patients ranges from 7 to 60% depending on patient characteristics, trauma severity and mechanism of injury [1]. Additionally, TE rates among patients with pelvic and acetabular fractures have been shown to reach up to 18% [2] and pulmonary embolism (PE) is a common cause of death within the first 24 h after trauma [3]. Injuries of the lower extremities and the pelvic region are associated with significantly higher rates of deep vein thrombosis (DVT) and PE compared to a general trauma population [4]. It has also been shown that severity of injury (according to Injury Severity Score, ISS), number of surgical procedures, occurrence of pelvic injury (according to Abbreviated Injury Scale, AIS ≥ 2) and certain medical conditions (i. e. diabetes, renal failure) constitute independent risk factors for the development of TE events [5].

So far, data on the relationship between pelvic and acetabular injuries and TE events in multiple injured patients are rare. The majority of the above stated literature is related to investigations in single pelvic or acetabular injury. This is why we proposed our study to investigate TE in multiple injured patients with focus on potential negative effects of concomitant pelvic or acetabular injuries. We therefore hypothesize that multiple injured patients with fractures of the pelvis/acetabulum develop more TE complications compared to the former stated TE rates in the literature and the risk for TE complications is dependent on pelvic/acetabular injury severity. Thus, a patient-tailored thromboprophylaxis could possibly be reasonable.

## Methods

### TraumaRegister DGU® (TR-DGU)

The TraumaRegister DGU® of the German Trauma Society (Deutsche Gesellschaft für Unfallchirurgie, DGU) was founded in 1993. The aim of this multi-center database is a pseudonymized and standardized documentation of severely injured patients. Data are collected prospectively in four consecutive time phases from the site of the accident until discharge from hospital: (A) Pre-hospital phase, (B) Emergency room and initial surgery, (C) Intensive care unit and (D) Discharge. The documentation includes detailed information on demographics, injury pattern, comorbidities, pre- and in-hospital management, course on intensive care unit, relevant laboratory findings including data on transfusion and outcome of each individual. The inclusion criterion is admission to hospital via emergency room with subsequent ICU/ICM care or reach the hospital with vital signs and die before admission to ICU. The infrastructure for documentation, data management, and data analysis is provided by AUC—Academy for Trauma Surgery (AUC—Akademie der Unfallchirurgie GmbH), a company affiliated to the German Trauma Society. The scientific leadership is provided by the Committee on Emergency Medicine, Intensive Care and Trauma Management (Sektion NIS) of the German Trauma Society. The participating hospitals submit their data pseudonymized into a central database via a web-based application. Scientific data analysis is approved according to a peer review procedure laid down in the publication guideline of TraumaRegister DGU®.

The participating hospitals are primarily located in Germany (90%), but a rising number of hospitals of other countries contribute data as well (at the moment from Austria, Belgium, Finland, Luxembourg, Slovenia, Switzerland, The Netherlands, and the United Arab Emirates). Currently, approx. 30,000 cases from more than 650 hospitals are entered into the database per year.

Participation in TraumaRegister DGU® is voluntary. For hospitals associated with TraumaNetzwerk DGU®, however, the entry of at least a basic data set is obligatory for reasons of quality assurance.

The present study is in line with the publication guidelines of the TR-DGU and registered as TR-DGU project ID 2020-035.

### Definition and documentation of thromboembolic events

Clinically relevant TE events included deep vein thrombosis (DVT), pulmonary embolism (PE), myocardial infarction (MI) and stroke. We grouped the observed TE events into either venous TE (DVT, PE) or arterial TE (MI, stroke). Thrombosis of a superficial vein or upper extremity is defined as “other”. The presence of an ongoing thromboprophylaxis at the timepoint of confirmed TE diagnosis is reported as either mechanical/chemical thromboprophylaxis, or both. TE events after hospital discharge are not part of the documentation of the TraumaRegister DGU® and therefore are not included in this study.

### Definition and documentation of fracture types, fracture severity and injury severity

To investigate the influence of injury severity of pelvic and acetabular fractures in multiple injured patients on TE complications the respective Abbreviated Injury Scores [6] (AIS, AIS_Pelvis_, AIS_Acetabulum_) and Tile/AO/OTA classification [7] of pelvic fractures were used. We performed a subgroup analysis of patients with additional lower extremity fractures (AIS_Lower Extremity_ = 2 or ≥ 3) to determine the effect of additional injuries to the lower extremity on TE rates. Overall injury severity was calculated according the Injury Severity Score (ISS) [8].

### Study Population, Inclusion and Exclusion Criteria

TraumaRegister DGU® data of patients with pelvic or acetabular fracture treated between 2010 and 2019 were analyzed. Patients were included in our analysis if records were complete regarding documentation of TE events. Consequently, basic data sets that do not report on TE events were not taken into account. The following exclusion criteria were used: age < 16 years, ISS < 16, death ≤ 24 h after admission, severe acute traumatic brain injury (AIS_Head_ ≥ 4) and patients treated in a different hospital prior to admission or transferred early (≤ 48 h after admission) to another hospital.

In order to control for some confounding factors for TE development such as anticoagulation, time of mechanical ventilation, ICU- and hospital length of stay, we excluded patients with severe acute brain injury (AIS_Head_ ≥ 4).

### Statistical analysis

Statistical analysis was performed with SPSS (IBM Inc., Version 24, Armonk, NY, USA). Continuous variables are shown as mean ± standard deviation (SD), incidence rates as percentages and distributed data as median and interquartile ratio (IQR), respectively. Differences in the study population were compared with the *χ*^2^-test for categorial variables and the Mann–Whitney *U*-test for continuous variables, respectively. A *p *value < 0.05 was considered significant. To identify independent risk factors for the development of TE complications a multivariable logistic regression analysis was used. The analyzed predictors are shown in Table 1. Odds Ratios (OR) are presented with 95% confidence intervals (CI_95_). Nagelkerke's *R*^2^ was used to describe the predictive power of the model.

**Table 1:** Demographics of patients with (TE) and without TE (Non-TE)

	Non-TE *n* = 10,113 (95.1%)	TE *n* = 521 (4.9%)	*p* value
Age (years; mean ± S.D.)	48 ± 19	53 ± 19	< 0.001*
Age ≥ 60 years, *n* (%)	2892 (28.6%)	203 (39.0%)	< 0.001*
Male sex, *n* (%)	6784 (67.1%)	371 (71.2%)	0.056
Concomitant diseases (ASA 3/4), *n* (%)	1052 (11.8%)	78 (17.5%)	0.001*
Blunt mechanism, *n* (%)	9556 (98.4%)	496 (98.4%)	0.95
*Accident mechanism*			
Car, *n* (%)	2676 (26.8%)	135 (26.0%)	0.71
Motorcycle, *n* (%)	1568 (15.7%)	91 (17.5%)	0.26
Bike, *n* (%)	522 (5.2%)	29 (5.6%)	0.71
Pedestrian, *n* (%)	1054 (10.5%)	59 (11.4%)	0.55
Fall from height (> 3 m), *n* (%)	2800 (28.0%)	141 (27.2%)	0.68
Low energy fall, *n* (%)	610 (6.1%)	24 (4.6%)	0.17
ISS (points; mean ± S.D.)	28 ± 10	32 ± 12	< 0.001*
AIS Head ≤ 3, *n* (%)	1801 (17.8%)	101 (19.4%)	0.36
AIS Thorax ≥ 3, *n* (%)	6318 (62.5%)	353 (67.8%)	0.015*
AIS Abdomen ≥ 3, *n* (%)	2390 (23.6%)	189 (36.3%)	< 0.001*
AIS Spine ≥ 3, *n* (%)	1241 (12.3%)	87 (16.7%)	0.003*
AIS Pelvis ≥ 3, *n* (%)	6007 (69.3%)	375 (72.0%)	0.19
AIS Extremities ≥ 3, *n* (%)	8137 (80.5%)	432 (82.9%)	0.17
Legs injured (AIS ≥ 2)	4607 (45.6%)	295 (56.6%)	< 0.001*
*Shock (BP* ≤ *90 mmHg)*			
Prehospital, *n* (%)	1464 (16.3%)	107 (24.0%)	< 0.001*
ER, *n* (%)	1587 (16.6%)	133 (27.5%)	< 0.001*
Organ failure, *n* (%)	4088 (43.2%)	331 (66.3%)	< 0.001*
Multi organ failure, *n* (%)	2405 (25.4%)	242 (48.4%)	< 0.001*
OF respiratory, *n* (%)	1775 (18.8%)	180 (36.1%)	< 0.001*
OF coagulation, *n* (%)	1693 (17.9%)	168 (33.7%)	< 0.001*
Sepsis, *n* (%)	812 (8.7%)	127 (26.0%)	< 0.001*
Number of operative procedures, mean, S.D	4.5 ± 5.8	8.0 ± 9.8	< 0.001*
Transfusion (y/n), *n* (%)	2408 (23.8%)	210 (40.3%)	< 0.001*
PRBC (n; mean ± S.D.)	1.4 ± 3.9	3.4 ± 7.9	< 0.001*
Infused volume prehospital (ml; mean ± S.D.)	943 ± 679	1046 ± 777	0.013*
Infused volume ER (ml; mean ± S.D.)	1729 ± 2000	2609 ± 2757	< 0.001*
Mechanical ventilation (days; mean ± S.D.)	5.1 ± 9.8	11.2 ± 15.4	< 0.001*
ICU LOS (days; mean ± S.D.)	11 ± 14	21 ± 23	< 0.001*
Overall hospital LOS (days; mean ± S.D.)	29 ± 22	42 ± 33	< 0.001*
In-hospital mortality, *n* (%)	486 (4.8%)	89 (17.1%)	< 0.001*

*S.D.* standard deviation, *ASA* Classification of American Society of Anesthesiologists, *ISS* Injury Severity Score, *AIS* Abbreviated Injury Scale, *BP* blood pressure, *OF* organ failure, *PRBC* packed red blood cells, *ER* emergency room, *LOS* length of stay

## Results

### Population

**Fig. 1:**
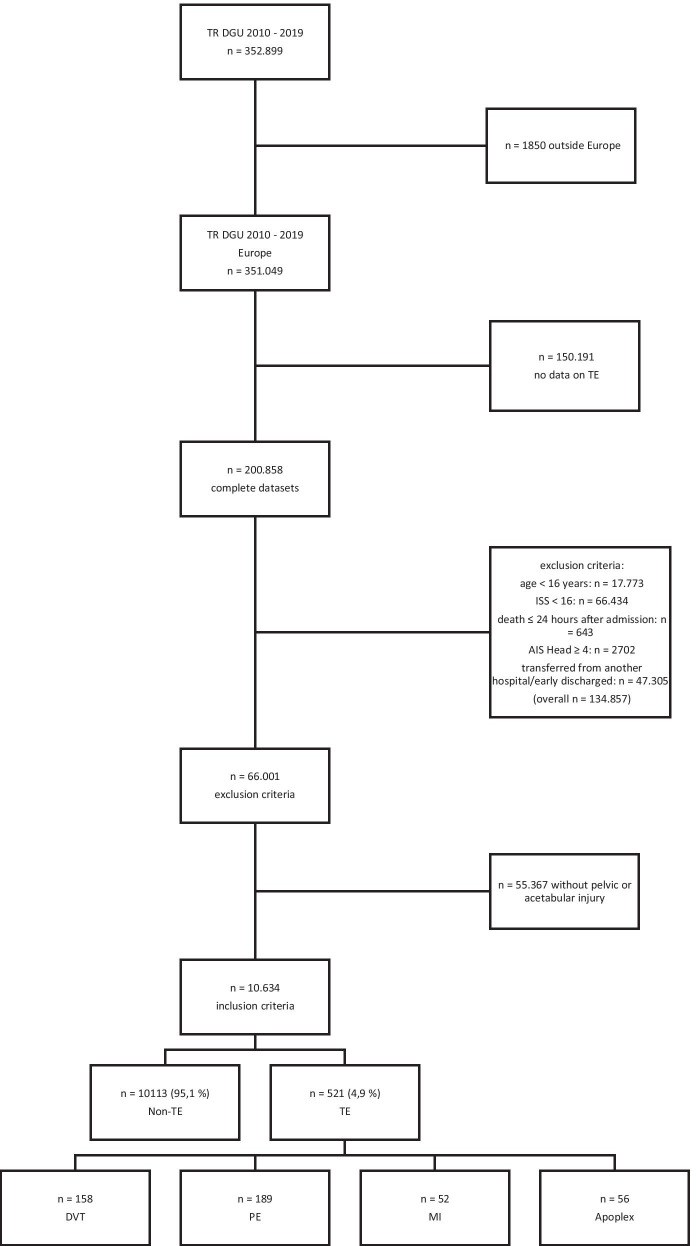
Inclusion process

The TraumaRegister DGU® (TR-DGU) consists of 353.899 datasets of patients documented between 2010 and 2019. After inclusion and exclusion criteria were applied, 10.634 complete datasets were enrolled in our final investigation (Fig. 1).

### Patient characteristics

Table 1 shows demographic characteristics of patients with (TE) and without TE (Non-TE) complications. Our study population is homogenous concerning sex and mechanism of injury. Significant differences are observed regarding age, pre-existing medical conditions, overall injury severity, thoracic and abdominal injuries as well as incidence of hemorrhagic shock and need for mass transfusion (> 10 PRBCs in the overall time course of treatment), appearance of complications such as organ failure and sepsis. No significant differences were observed among patients with and without TE complications regarding the incidence of severe pelvic or acetabular fracture as well as lower extremity injuries. P-values for each single parameter are documented in Table 1.

Patients suffering from thromboembolic events had higher morbidity and mortality rates. Overall mortality was 17.1% for the TE-group compared to 4.8% in the group without thromboembolic events (*p* < 0.001). Duration of mechanical ventilation, days spent in ICU as well as overall hospital length of stay were significantly higher among patients suffering from TE compared to patients without a thromboembolic complication (mechanical ventilation: 11.2 ± 15.4 vs. 5.1 ± 9.8 days; ICU: 21 ± 23 vs. 11 ± 14 days; overall hospital length of stay: 42 ± 33 vs 29 ± 22 days; all *p* < 0.001).

### Thromboembolic events

**Fig. 2:**
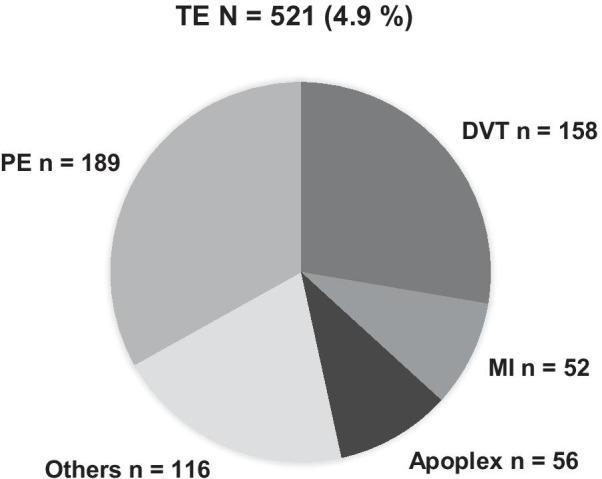
Incidences of thromboembolic events (TE, *n* = 521). *DVT* Deep Vein Thrombosis, *PE* Pulmonary Embolism, *MI* Myocardial Infarction

521 of 10.634 patients suffered from a thromboembolic event (4.9%). PE accounted for 1.8% and DVT for 1.5% of cases, respectively. TE incidences are shown in Fig. 2. At the timepoint of TE diagnosis 502 of 521 patients (96.4%) were either under mechanical and/or pharmacological thrombosis prophylaxis.

To evaluate a possible thrombogenic effect of additional injuries of the lower extremities among patients with pelvic/acetabular fractures we analyzed TE rates of a subgroup of patients with additional lower extremity trauma defined as AIS_Lower Extremity_ = 2 or ≥ 3 (Table 2).

**Table 2:** Incidence of thromboembolic events (TE), venous TE and arterial TE among patients with additional lower extremity trauma defined by AIS

	Without lower extremity trauma* (*n* = 5732)	Additional AIS_Lower Extremity_ 2 (*n* = 1812)	Additional AIS_Lower Extremity_ ≥ 3 (*n* = 2795)	*p *value
TE	226 (3.9%)	105 (5.5%)	190 (6.4%)	< 0.001
Venous TE	131 (2.3%)	73 (3.8%)	118 (4.0%)	< 0.001
Arterial TE	61 (1.1%)	17 (0.9%)	29 (1.0%)	0.559

*AIS* Abbreviated Injury Scale

*p* value indicating difference in TE between patients without and additional lower extremity trauma (AISLower Extremity ≥ 2)

*AIS 0 or 1

Patients suffering from combined pelvic/acetabular and lower extremity trauma (AIS_Lower Extremity_ ≥ 2) have a significantly higher overall chance to develop TE events (*p* < 0.001). In a subsequent evaluation of different types of TE we were able to show significant differences for development of venous TE (*p* < 0.001) but not for arterial TE (*p* = 0.778) complications.

### Influence of pelvic/acetabular injury severity on thromboembolic events

**Fig. 3:**
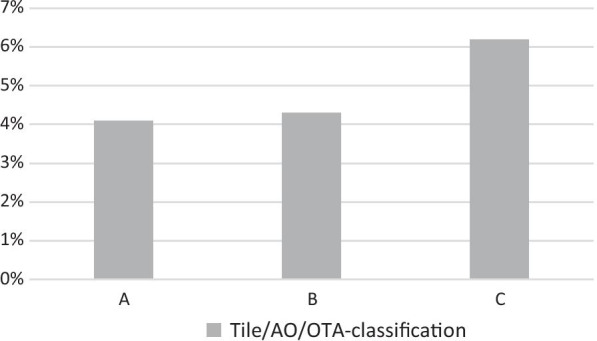
Incidence of thromboembolic events depending on injury severity of pelvic fractures according to Tile/AO/OTA-classification

The influence of pelvic and acetabular fracture severity on the occurrence of TE complications among multiply injured patients was examined using AIS_Pelvis_, Tile/AO/OTA-classification [7] and AIS_Acetabulum_. Table 3 demonstrates the relationship between AIS_Pelvis_ and the incidence of TE events in multiple injured patients. Increasing injury severity and fracture complexity, as represented by AIS_Pelvis_, was associated with a significant increase in thromboembolic events (*p* < 0.001). This is similar when TE rates of patients with pelvic injury are compared to fracture severity/instability according to the Tile/AO/OTA-classification of pelvic fractures (Fig. 3). TE incidences for type A fractures were 4.1%, type B 4.3% and type C 6.2%, respectively. The Chi^2^-test for linear trend shows an increase of TE rates with increasing fracture severity and instability of pelvic fractures represented by the Tile/AO/OTA-classification (*p* < 0.001).

**Table 3:** Total number and incidence of thromboembolic events depending on injury severity of pelvic fractures according to AIS_Pelvis_ (Chi^2^-test for linear trend < 0.001)

Severity of pelvic injury	Non-TE	TE	Overall
AIS 2	3106 (95.5%)	146 (4.5%)	3252 (30.6%)
AIS 3	2496 (96.4%)	93 (3.6%)	2589 (24.3%)
AIS 4	3363 (94.7%)	189 (5.3%)	3552 (33.4%)
AIS 5	1148 (92.5%)	93 (7.5%)	1241 (11.7%)

*AIS* Abbreviated Injury Scale

**Fig. 4:**
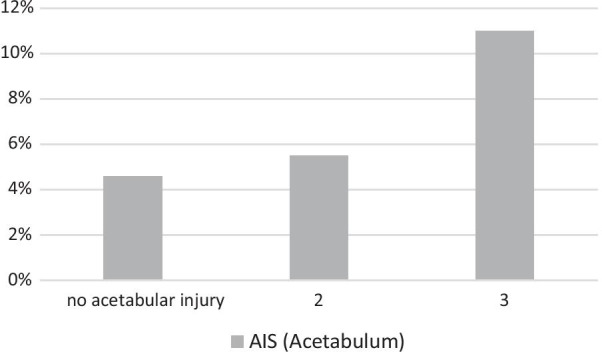
Incidence of thromboembolic events depending on injury severity of acetabular fractures according to AIS_Acetabulum_; *AIS* Abbreviated Injury Scale

Multiple injured patients without injury to the acetabulum have TE rates of 4.6% compared to 5.5% for multiple injured patients with AIS_Acetabulum_ = 2 and 11.0% with AIS_Acetabulum_ = 3, respectively (Fig. 4). The Chi^2^-test for linear trend was significant (*p* < 0.019).

### Influence of number of operative procedures on thromboembolic events

**Fig. 5:**
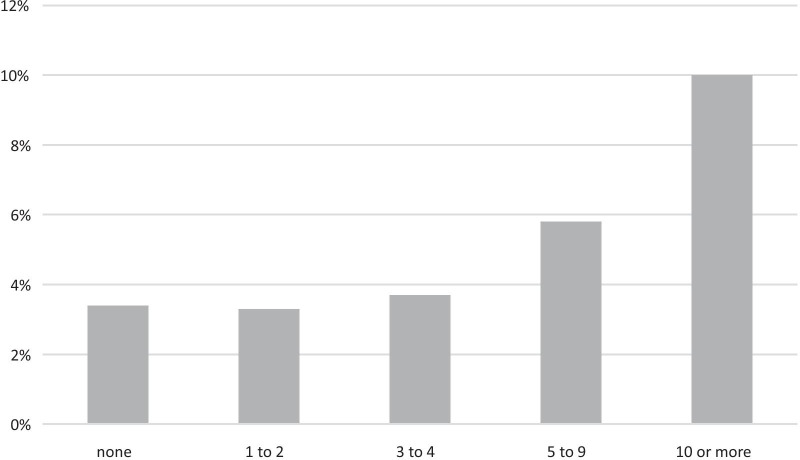
Influence of number of operative procedures on thromboembolic events

8935 (84.02%) of 10.634 included patients had surgical treatment. Non-surgical treatment was performed in 16.2% of the Non-TE group compared to 11.1% in the TE group. TE rates in patients treated non-operatively were 3.4%. This was found as well, when total numbers of surgical procedures or grouped counts of surgeries (Fig. 5) were analyzed. Increasing numbers of additional procedures were significantly associated with the chance to develop TE complications (1–2 surgeries: 3.3%; 3–4: 3.7%; 5–9: 5.8%; ≥ 10: 10.4%).

### Multivariate logistic regression model

Univariate analysis documented certain risk factors increasing the chance for the development of thromboembolic events in multiple injured patients and pelvic/acetabular fractures. To determine whether the above identified factors (i. e. age, sex, ISS, organ specific AIS, fracture type, number of surgery procedures, transfusion characteristics, sepsis) serve as independent risk factors for TE development a multivariate logistic regression model with TE complication as dependent variable was conducted among *10.626* datasets. The results are shown in Table 4.

**Table 4:** Results of multivariate logistic regression model (*n* = 10.626)

	Odds ratio	95% CI	*p *value
Age ≥ 65 years	1.70	1.38–2.09	< 0.001*
Male sex	1.21	0.99–1.48	0.065
ISS ≥ 25	1.09	0.85–1.40	0.50
AIS Head 3	1.02	0.80–1.29	0.90
AIS Thorax ≥ 3	1.09	0.88–1.35	0.44
AIS Abdomen ≥ 3	1.38	1.11–1.71	0.004*
*Lower extremity trauma (reference: none)*			0.58
AIS 2	1.13	0.88–1.46	0.33
AIS ≥ 3	1.10	0.87–1.39	0.42
*Tile/AO/OTA-classification (Ref: Tile A)*			0.24
Tile B	1.03	0.79–1.34	0.83
Tile C	1.27	0.96–1.67	0.099
*AIS Acetabulum*			0.125
AIS 2	1.21	0.95–1.54	0.122
AIS 3	1.84	0.84–4.04	0.126
*Number of operative procedures (reference: 0)*			< 0.001*
1–2	0.98	0.70–1.36	0.89
3–4	0.96	0.67–1.37	0.82
5–9	1.34	0.95–1.87	0.092
≥ 10	1.98	1.36–2.86	< 0.001*
Sepsis	2.45	1.95–3.08	< 0.001*
*PRBC administered*			0.008*
1–9 units	1.12	0.19–1.41	0.34
Mass transfusion ≥ 10 units	1.72	1.22–2.42	0.002*

Nagelkerke’s *r*^2^ = 0.071

*Ref* reference category, *AIS* Abbreviated Injury Scale, *ISS* Injury Severity Score, *PRBC* packed red blood cell, *CI* confidential interval

* indicates significant results

Independent risk factors for the development of TE were sepsis, ≥ 10 operations, mass transfusion (≥ 10 PRBCs), age ≥ 65 years and AIS_Abdomen_ ≥ 3 (all *p* < 0.001). No correlation was found for overall injury severity (ISS), moderate traumatic brain injury (AIS_Head_ = 3), additional injury to lower extremities, type B and C pelvic fracture according to Tile/AO/OTA, closed (AIS_Acetabulum_ = 2) or open acetabular fracture (AIS_Acetabulum_ = 3).

## Discussion

In this study we sought to identify thrombogenic risk factors and predictors among multiple injured patients with pelvic and/or acetabular fractures.

Available data regarding TE incidences among trauma patients are inconsistent. These numbers strongly depend on the respective trauma patient collective, including injury mechanisms as well as injury severity. In a retrospective study from the National Trauma Data Bank (NTDB) of the American College of Surgeons incidences for DVT and PE were 1.06% and 0.42%, respectively [9]. Paffrath et al. [5] documented venous TE rates of 1.8% among 7937 trauma patients with an ISS ≥ 9 and Lichte et al. [4] published overall TE rates of 2.8% among 40.846 polytrauma patients. In comparison, the incidence of thromboembolic events in our overall collective comprising multiple injured patients with ISS ≥ 16 and fractures of the pelvis and/or acetabulum was 4.9%, which states a considerable higher incidence.

To further appraise our higher rate of TE events in multiple injured patients one can compare TE rates in trauma patients with isolated pelvic / acetabular fractures. Patients with isolated fractures of the pelvis or acetabulum are also at high risk of developing a thromboembolic complication [3, 10–13] since these fractures are often associated with injuries to pelvic and sacral vessels and often require longer times of immobilization and bed rest [10, 11]. Moed et al. [12] retrospectively analyzed 13.589 patients with pelvic and acetabular fractures that were treated surgically and documented 113 (0.83%) TE events (DVT: 0.51%; PE: 0.21%; both: 0.12%). Kim [13] performed a scheduled CT-venography 7–14 days after trauma in 55 patients with fracture of the pelvis and 40 patients with acetabular fracture. In his study 32 (33.7%) of 95 patients developed a venous TE complication, PE was present in 9 cases (9.5%). In a similar study, Sen et al. found overall rates for TE events of 28.6% (DVT: 21.4%; PE: 17.9%). Some literature even reports rates for DVT of up to 61% and 10% for PE among traumatized patients with pelvis and/or acetabular fractures [3]. The major difference between their studies and ours is that we report on clinically suspected and then diagnostically confirmed TE complications, while the former stated studies explicitly looked for thromboembolism with scheduled radiographic exams. In light of this, TE events in patients with pelvic / acetabular fracture seem to be more common than it appears and could be a reason for the higher incidence of TE complications in our study in comparison to other investigations of severely injured patients.

It has been widely published that patients with pelvic trauma are at high risk to develop thromboembolic complications and e.g. age, prolonged immobilization and excessive blood transfusion are further contributing factors [14, 15]. Thromboprophylaxis is invariably recommended by guidelines worldwide in the treatment of severely injured patients or trauma patients taken care of in an ICU. In the absence of contraindications (e.g. active bleeding, relevant traumatic brain injury) early pharmacologic thromboprophylaxis is recommended over the use of mechanical prophylaxis (e.g. intermittent pneumatic compression). The routinely use of inferior vena cava filters for thromboprophylaxis is not recommended in the respective guidelines and the placement of this device is rather reserved for special circumstances [16–20]. Since the vast majority of patients included in our analysis were treated in German hospitals we can only assume a pharmacological treatment according to the German guideline [20], because details beyond the stated presence or absence of mechanical/pharmacological prophylaxis are not recorded in the TR-DGU. The adherence to current guidelines in our cohort is supported by the fact that 96.4% of patients suffering from TE received prior thromboprophylaxis treatment. This is in line with a recent survey on venous thromboembolism prophylaxis after pelvic and acetabular fractures where—although guidelines on thromboprophylaxis exist—no consensus was reported on the actual treatment practices [21].

Published data suggest that TE development is associated with increasing age. In a study by Fuchs et al. [22] age > 40 years was identified as an independent risk factor for thromboembolic complications. Similar, significantly higher numbers of TE events in patients aged > 60 years were reported in a study by Lichte et al. [4] Further studies confirmed the relationship between TE complications and increasing age [1, 9, 23, 24]. In line with the current literature we identified age > 65 years as an independent risk factor for TE complications in multiple injured patients with fractures of the pelvis and/or acetabulum.

Previous studies have highlighted the influence of certain underlying medical conditions on the development of thromboembolic complications. Especially pre-existing cardiac, respiratory or musculoskeletal disorders place trauma patients at higher risk for complications, i.e. thromboembolism. In the study by Paffrath et al. [5], underlying medical disorders such as diabetes, renal failure, malignancies, congenital or acquired coagulation disorders were independently associated with thromboembolic events. Our findings support the current evidence. Patients with relevant pre-existing medical disorders as defined by American Anesthesiologist`s ASA score of 3 or 4 were more likely to develop a TE complication in our study. Possibly, establishing risk scores and identifying trauma patients at higher risk for TE development could lead to a more individual and patient-tailored antithrombotic therapy.

In our descriptive analysis higher numbers of TE complications were associated with increasing injury severity (ISS) and injuries to the trunk (thorax, abdomen, spine). Our multivariate logistic regression model revealed (severe) abdominal injury (AIS_Abdomen_ ≥ 3) as an independent risk factor for the occurrence of thromboembolism. The relevance of overall injury severity as a risk factor for TE complications has been widely studied [5, 25–28]. In contrast, sufficient evidence on different injury patterns or injury localizations associated with higher thrombogenic risk among multiple injured patients is missing. Data suggesting higher thromboembolic risk exists for thoracic, spinal, abdominal, pelvic and lower extremity trauma as well as penetrating injuries in polytrauma patients [4, 9, 25, 28–33]. Our data suggest higher TE rates associated with injuries to the thorax, abdomen and spine in multiple injured patients with pelvic or acetabular injury. However, in our collective, multivariate logistic regression found that severity of pelvic or acetabular injury is not an independent risk factor for TE complications in multiple injured patients with pelvic or acetabular injury. This is true despite our significantly higher rates of TE complications in patients suffering from more than serious acetabular injury (AIS_Acetabulum_ ≥ 3) or more then severe pelvic injuries (AIS_Pelvis_ ≥ 4). One reason and possible explanation might be that these patients also suffer from relevant hemorrhage, coagulopathy, which already proved to be an independent risk factors for TE complications in our study cohort. Also, supposedly, patients with trauma to the trunk are more likely to suffer from TE events because of higher numbers of hemorrhage in that cohort, increasing number of operative procedures and longer time of immobilization [4, 9, 25, 28–33]. As hemorrhage, extensive surgery and sepsis are common complications in patients with abdominal injury, these patients pose an intensive thrombogenic risk [1]. As expected, neither in our univariate nor the multivariate analysis moderate traumatic brain injury had an impact on thromboembolic complications. These finding are in line with the current literature [34].

Multiple traumatized patients with pelvic/acetabular fractures often suffer from internal or external bleeding and hemorrhagic shock [5, 10, 35]. Hence, resuscitation often requires transfusion of high amounts of blood products [4, 5]. In the present study either the presence of hemorrhagic shock or the need for mass transfusion (≥ 10 PRBCs) significantly increased the thromboembolic risk.

Differences concerning thromboembolic complications with regard to pelvic and acetabulum fracture type or severity are little investigated in the literature. Our univariate analysis revealed higher TE rates with increasing fracture complexity of pelvic/acetabular injuries. In contrast, multivariate analysis did not identify pelvic and acetabular fractures as independent risk factors for TE development. Bagaria [36] observed that injuries to posterior structures in type B and C pelvic fractures are associated with kinking of iliac and femoral vessels thus, supposedly, promoting DVT and PE. In a study by Kim et al. [13], vertical sheer type injuries according to the Young-Burgess classification were associated with significantly higher chance for thromboembolism when compared to anterior compression type and lateral compression type pelvic injury. When the relationship between thromboembolism and acetabular fracture was examined, dominantly posterior localized fracture types according to Judet-Letournel were associated with higher TE rates. Similar results were reported by Sen et al. [11] with higher TE numbers related to increasing fracture instability and complexity of pelvic and acetabular fractures. Our results are in line with this literature showing higher thromboembolic risk with increasing injury severity of pelvic and acetabular fractures (according to AIS_Pelvis_ and AIS_Acetabulum_) as well as fracture instability of pelvic injuries (according to Tile/AO/OTA-classification). As shown, especially pelvic fractures with AIS_Pelvis_ ≥ 4/Tile C as well as severe acetabular fractures with AIS_Acetabulum_ = 3 represent a group of fractures in patients with mainly high-energy injury mechanism, highly unstable fracture situation, high percentage of hemorrhages, high numbers of required operative procedures and prolonged time of immobilization. Multiple injured patients with high-energy injury mechanism, injuries to predominantly posterior pelvic and acetabular structures, relevant blood loss and the need for repetitive operative treatment are at high risk for the development of thromboembolic complications.

Interestingly, in our multivariate logistic regression analysis injury severity of pelvic or acetabular fracture was no independent risk factor for TE complication. Our explanation for this finding is that acetabular/pelvic injury increases TE risk as shown above when our multiple injury patient collective with mandatory pelvic/acetabular fractures has been compared to published research with overall multiple injured patient studies. In multiple injured patients with acetabular or pelvic injuries the severities of these injuries seem to have no further impact on thromboembolic risk. Importantly, in this context, more relevance seems to lay on risk factors like hemorrhage, mass transfusion, sepsis, abdominal injury etc. that have an imminent influence to exaggerate TE development in these patients. Further research might suggest adapted thrombosis prophylaxis or the establishment of screening examinations in this group of patients. This is somewhat supported by Lowe et al. who found relevant incidences of venous thromboembolism in pelvic and lower extremity trauma despite adherence to modern venous thromboembolism prophylaxis protocols [37].

The probability of developing a thromboembolic event increases with the number of operative procedures among trauma patients. The present study suggests an increased risk for the development of a TE complication among multiple injured patients with pelvic and acetabular fractures with increasing numbers of surgical procedures performed in the course of treatment. When ≥ 10 operations were required, patients were under significantly higher risk to suffer from thromboembolic complications. Pathophysiological mechanisms that drive prothrombotic factors in patients with multiple surgeries remain unclear. Evidence exists in trauma patients that, on a molecular level, a procoagulatory metabolic state (acute traumatic coagulopathy) caused by the initial trauma is intensified by repetitive surgical trauma [13, 38–40]. Furthermore, patients requiring multiple operative procedures might have longer times of immobilization, mechanical ventilation and lCU-length of stay that are also associated with TE development [4, 5, 22, 24, 32].

Thromboembolic events among multiple injured patients are a devastating complication during recovery. When compared to Non-TE patients, patients suffering from thromboembolic complications had significantly higher mortality and morbidity in our investigation. Besides the acute injury, complications during the course of treatment promote the development of thromboembolism. This is especially true for septic complications. Our data suggest sepsis to be a major prothrombotic factor in polytraumatized patients with 26.5% compared to 8.7% of patients suffering from sepsis in the TE-group and Non-TE-group, respectively. Multivariate regression analysis revealed sepsis as an independent risk factor for development of thromboembolism. In a study by Paffrath et al. [5], presence of sepsis almost tripled the chance to suffer from TE complications. Other authors report similar results identifying septic complications as key factor for thromboembolic events [41, 42].

The current study has several limitations. Clinically inapparent thromboembolism and thromboembolism diagnosed after hospital discharge were not part of the documentation of the TraumaRegister DGU®. Therefore documented incidences represent only clinically relevant TE complications. Supposedly, the incidence of thromboembolic events in our study population might be underestimated. Furthermore, no information can be given regarding advantages or disadvantages of different mechanism or substances of thromboprophylaxis as only the presence or absence of mechanical/pharmacological prophylaxis is recorded. This is why we are not able to comment on the type or substance administered for pharmacological prophylaxis or the mechanical device used. Additionally, we cannot report on efforts made to evaluate coagulability (e.g. thrombelastography) or to ascertain thromboprophylaxis (e.g. anti-Xa monitoring), since these parameters were not documented in the TR-DGU. On the other hand, measuring anti-Xa or thrombelastography for thromboembolic risk estimation is not part of the routine [20]. Types of acetabular fractures are only differentiated in either “none”, “closed” (AIS_Acetabulum_ = 2) or “open” (AIS_Acetabulum_ = 3). The high variability of fracture morphology as described in the classification of acetabular fractures by Judet and Letournel is therefore not documented in the TraumaRegister DGU® so that our reported results concerning acetabular fractures have to be interpreted accordingly.

## Conclusion

Multiple injured patients with ISS ≥ 16 suffering from pelvic and/or acetabular fractures are at high risk of developing thromboembolic complications. Additional fractures to the lower extremities potentially intensify this effect. Independent risk factors for the development of thromboembolic events in our study cohort were age ≥ 65 years, mass transfusion (≥ 10 PRBCs), AIS_Abdomen_ ≥ 3, sepsis and ≥ 10 operative procedures. Thus, in our cohort of multiple injured patients with acetabular or pelvic injuries the severities of these injuries seem to have no further impact on thromboembolic risk. Importantly, in this context, more relevance seems to lay on the above-mentioned risk factors that have an imminent influence to exaggerate TE development in these patients. In conclusion, our study highlights the major impact of early hemorrhage, septic complications and abdominal injury on thromboembolic risk in severely injured trauma patients.

Further research among multiple injured patients is needed to ascertain predictors and patients at risk for thromboembolic complications which might possibly lead to individualized screening examinations and a patient-tailored, intensified, thromboprophylaxis.

## Data Availability

The authors state that the sensitive data presented in this study is available from a third party, which is the AUC—Academy for Trauma Surgery („Akademie der Unfallchirurgie GmbH“), which is the owner of the data of the TR-DGU. The data underlying the results presented in the study are available from: AUC—Akademie der Unfallchirurgie GmbH, Wilhelm-Hale-Straße 46b, 80639 München, Deutschland, Tel.: +49221 888239-10, Email: support-tr@auc-online.de, http://www.traumaregister-dgu.de. All data presented is approved according to a peer review procedure laid down in the publication guideline of TraumaRegister DGU®.

## Abbreviations

AIS: Abbreviated Injury Scale
AO: Arbeitsgemeinschaft für Osteosynthesefragen
ASA: American Society of Anesthesiologists
BP: Blood pressure
DGU: Deutsche Gesellschaft für Unfallchirurgie
DVT: Deep vein thrombosis
ER: Emergency Room
ICU: Intensive care unit
ISS: Injury Severity Score
LOS: Length of Stay
MI: Myocardial infarction
OF: Organ Failire
OTA: Orthopedic Trauma Association
PE: Pulmonary embolism
PRBC: Packed red blood cells
TE: Thromboembolic
TR-DGU: TraumaRegister DGU®
